# The appearance of mimetic *Heliconius* butterflies to predators and conspecifics

**DOI:** 10.1111/evo.13583

**Published:** 2018-09-05

**Authors:** Denise Dalbosco Dell'Aglio, Jolyon Troscianko, W. Owen McMillan, Martin Stevens, Chris D. Jiggins

**Affiliations:** ^1^ Butterfly Genetics Group, Department of Zoology University of Cambridge United Kingdom; ^2^ Smithsonian Tropical Research Institute Panama City Panama; ^3^ Centre for Ecology and Conservation, College of Life and Environmental Sciences University of Exeter Penryn United Kingdom

**Keywords:** Aposematism, avian vision, butterfly vision, mating behavior, opsin, UV light

## Abstract

Adaptive coloration is under conflicting selection pressures: choosing potential mates and warning signaling against visually guided predators. Different elements of the color signal may therefore be tuned by evolution for different functions. We investigated how mimicry in four pairs of *Heliconius* comimics is potentially seen both from the perspective of butterflies and birds. Visual sensitivities of eight candidate avian predators were predicted through genetic analysis of their opsin genes. Using digital image color analysis, combined with bird and butterfly visual system models, we explored how predators and conspecifics may visualize mimetic patterns. Ultraviolet vision (UVS) birds are able to discriminate between the yellow and white colors of comimics better than violet vision (VS) birds. For *Heliconius* vision, males and females differ in their ability to discriminate comimics. Female vision and red filtering pigments have a significant effect on the perception of the yellow forewing band and the red ventral forewing pattern. A behavioral experiment showed that UV cues are used in mating behavior; removal of such cues was associated with an increased tendency to approach comimics as compared to conspecifics. We have therefore shown that visual signals can act to both reduce the cost of confusion in courtship and maintain the advantages of mimicry.

In many species, natural and sexual selection has tuned sensory systems to detect specific and biologically relevant signals (Stevens 2013; Cronin et al. 2014). Many signals reflect a balance between the strength of sexual selection and the pressure of predation. One of the most widely studied sensory modalities is vision and color, which is influenced by both mate choice and predation (Endler 1980). The traditional view is that if predation is a relatively stronger selective force than sexual selection, coloration will be more conspicuous for aposematic species or more cryptic for camouflaged species. In contrast, if predation is relatively weaker, color patterns will be closer to the optimum for mate choice (Endler 1978, 1992).

Conflicts between of natural and sexual selection can also affect the wing pattern colouration of mimetic butterflies, as communication is aimed both toward their own species and to predators. This is particularly true in *Heliconius* butterflies, in which individuals of two or more chemically defended species share a mutual selective benefit from shared color patterns (Brown 1981; Mallet 1999). Bright and conspicuous patterns warn that butterflies are toxic (Engler‐Chaouat and Gilbert 2007) and bird predators learn to avoid these unpalatable butterflies (Chai 1986; Pinheiro 2003; Langham 2004). Within a given region, many different butterfly species have identical patterns due to Müllerian mimicry (Müller 1879). *Heliconius* butterflies also find and choose potential mates based on color signals (Jiggins et al. 2001; Sweeney et al. 2003; Kronforst et al. 2006). Thus, predator selection favors convergence on identical wing patterns, while sexual selection favors pattern differences that allow individual butterflies to distinguish between conspecifics and heterospecifics.

To understand the trade‐off between using color patterns as signals to predators and conspecifics, we need to consider the appearance of butterflies from both a bird and butterfly visual perspective. Avian predators detect light in the ultraviolet (UV) range, and diurnal birds, which likely have excellent color vision, broadly fall into two different classes of color vision: a violet sensitive (VS) and an ultraviolet sensitive (UVS) group (Bennett and Cuthill 1994; Ödeen and Håstad 2013). Although most diurnal birds are thought to be sensitive to UV light to some degree, small differences between VS and UVS systems can produce large variation in the perception of colors in this part of the spectrum (Ödeen et al. 2012). Unfortunately, there is little data on the visual ability of tropical birds that are thought to be important predators and agents of selection of butterflies, such as Jacamars (Galbulidae) and flycatchers (Tyrannidae) (Chai 1986; Pinheiro 1996).

*Heliconius* butterflies also have well‐developed color vision. Indeed, color discrimination can be excellent in butterflies and there is potential for hidden channels of communication that could be used in mate selection, in which signals are not detected by predators. Since the discovery of an additional UV opsin and UV‐reflecting yellow wing pigment in the *Heliconius* lineage, it has been suggested that UV‐based signals could facilitate species‐specific recognition while not compromising Müllerian mimicry (Briscoe et al. 2010; Finkbeiner et al. 2017). This could enable differences in butterfly color patterns to arise under sexual selection, while at the same time maintaining the similarity of comimic pairs to avian vision.

In *H. erato*, two UV opsins confer sensitivity to ∼355 nm (UVRh1) and ∼398 nm (UVRh2), and it has been suggested that this might allow a greater degree of discrimination of yellow patches (Briscoe et al. 2010; Bybee et al. 2012). The compound eye of *H. erato* is sexually dimorphic and males express only UVRh2 while females express both UV opsins in separate photoreceptors (McCulloch et al. 2016, 2017). Differences in photoreceptor ratios therefore might play a role in sexual selection and identification of mates. Such a communication system may allow more refined visual discrimination of UV signals than afforded by avian vision, which involves just one UV sensitive receptor.

The role of color in mate choice in butterflies has been frequently investigated, and a few studies have evaluated preferences for variation in UV reflectance. For example, UV brightness is a strong component of male attractiveness in both *Colias* and *Eurema* butterflies (Silberglied and Taylor Jr. 1973; Rutowski et al. 2007; Kemp 2008) while *Polymmatus icarus* males prefer UV‐absorbing females (Knüttel and Fiedler 2001). The use of UV signals in signaling between different *Heliconius* species to avoid mating confusion has not been addressed. In *H. erato*, both the UV and long wavelength component of the yellow band contribute to the signal used for conspecific recognition (Finkbeiner et al. 2017).

Previous studies have addressed the fidelity of mimicry and the ability of predator and butterflies to distinguish between mimics in different genera (Llaurens et al. 2014; Su et al. 2015; Mérot et al. 2016; Thurman and Seymoure 2016). Here, we address a different question: what is the ability of individuals to distinguish between sympatric comimic species? This is a common challenge faced by many *Heliconius* to find the right mate, although all use the same pigment molecules to make their mimetic colours (Nijhout and Wray 1988). It has been shown that closely related mimics often demonstrate signal confusion during courtship due to their similar appearances (Jiggins et al. 2001; Estrada and Jiggins 2008). For example, *Heliconius erato* males use wing colour pattern in mate recognition and are more likely to approach and court with models of their own coloration (Estrada and Jiggins 2008). Recently, it was discovered that *Heliconius* adults have a high number of chemosensory genes (Briscoe et al. 2013), and females use chemical signals to select conspecific males (Darragh et al. 2017). *Heliconius* butterflies are therefore a useful system for investigating the conflicting selection pressures of predation and mate preference.

In this study, we examine the coloration of *Heliconius* comimic pairs and investigate visual signaling relevant to mimicry both from the perspective of butterflies and birds. Here, we aimed to: (1) investigate the visual pigments of potential avian predators determined from amino acid sequences; (2) analyze variation in color between four comimic pairs to estimate the capacity of *Heliconius* butterflies and birds to effectively perceive the differences within and between mimetic species, using digital photography; (3) use behavioral tests to explore whether UV reflectance might be important for recognition of conspecifics. These data are used to test the hypothesis of cryptic channels of communication between butterflies, which would reduce the cost of confusion in courtship while still maintaining the advantages of Müllerian mimicry against predation.

## Material and Methods

### AVIAN PREDATOR VISION

Eight species of bird were selected to ascertain the visual system of potential *Heliconius* predators: white‐whiskered puffbird (*Malacoptila panamensis*), blue‐crowned motmot *(Momotus momota*), rufous‐tailed jacamar (*Galbula ruficauda*), black‐tailed trogon (*Trogon melanurus*), slaty antshrike (*Thamnophilus atrinucha*), great kiskadee (*Pitangus sulphuratus*), ochre‐bellied flycatcher (*Mionectes oleaginous*), and Panama flycatcher (*Myiarchus panamensis*). Although not all of these species are known to feed on butterflies, all occur near the study site in Panama, are mainly insectivorous, and most show the “sit‐and‐wait” foraging behavior of capturing insects during flight.

The tissue samples used were obtained at the Smithsonian Tropical Research Institute Cryological Collection in Panama (See Table S1 for biorepository ID). Total DNA was extracted from muscle tissue with the DNeasy Blood and Tissue Kit (QIAGEN) using standard procedures. The difference between two types of bird visual system is the sensitivity of their short‐wavelength sensitive type 1 pigment (SWS1), which is shifted from ultraviolet to violet by amino acid replacements at the sites 84–94 (Ödeen and Håstad 2003). The primers used amplified a gene fragment coding the specific sites located in the SWS1 opsin (Ödeen and Håstad 2003; Bloch 2015).

PCR was conducted on a G‐Storm cycler (Somerton, UK). Each 20 μl reaction volume contained 2 μl total DNA extracts, 1 × BIOTaq DNA‐polymerase (Bioline), 2 μl 10 × NH4 reaction buffer, 1 μl of each primer, 0.2 mM of each dNTP, 0.8 μl 50 mM MgCl2 and 0.6 μl DMSO. Reaction conditions were 120 s at 94°C, 4 × (20 s at 94;°C, 20 s at 62°C, 10 s at 72°C), 6 × (20 s at 94°C, 20 s at 60°C, 11 s at 72°C), 30 × (20 s at 94°C, 20 s at 57°C, 12 s at 72°C) and 10 min at 72°C. In case of amplification of multiple products, the product was purified from a 1.5% agarose gel using MinElute Gel Extraction Kit (QIAGEN). PCR products were cleaned using the ExoSAP‐IT system (USB, Cleveland, Ohio) on 30 min at 37°C and 15 min at 80°C. PCR products were sequenced and DNA sequences were translated into amino acids to access the predicted type of spectral tuning of each species following Wilkie et al. (2000).

### STUDY SPECIES AND IMAGE COLLECTION IN DARK ROOM

Four pairs of *Heliconius* mimics that live in sympatry were selected for this study. The specimens were selected from the available collection of *Heliconius* butterfly wings in the Butterfly Genetics Group, Cambridge, UK. The comimic pairs were *H. erato lativitta* and *H. melpomene malleti* (*Hel/Hmm*; *n* = 14), *H. erato notabilis* and *H. melpomene plesseni* (*Hen/Hmp*, *n* = 10) collected in Ecuador, *H. erato demophoon* and *H. melpomene rosina* (Hed/Hmr, *n* = 10), *H. sapho* and *H. cydno* (Hs/Hc, *n* = 10) collected in Panama (Fig. S1).

Coloration was investigated using digital photography, following the methodology described recently and using an image analysis toolbox released for the programme Image J (Stevens et al. 2007; Troscianko and Stevens 2015). Dorsal and ventral wings of each specimen were photographed with a Fujifilm IS Pro UV‐sensitive digital camera with a quartz CoastalOpt UV lens (Coastal Optical Systems), fitted with a UV/IR blocking filter (Baader UV/IR Cut filter; transmitting between 400 nm and 680 nm) and a UV pass filter (Baader U filter; transmitting between 320 nm and 380 nm). The spectral sensitivity of the camera sensors was derived prior to photography (Stevens et al. 2007; Troscianko and Stevens 2015). Two photographs were taken in sequence, one in human‐visible spectrum and other in UV spectrum with the respective filters (Fig. S1). The photography setup used for the experiments consisted of a sheet of black ethylene‐vinyl acetate (EVA) used as a low‐UV reflective background, including a 40% gray standard (Spectralon® Labsphere) used for calibration. All the photographs were taken in constant light conditions, in a dark room with an UV/white broad emission spectrum light bulb simulating D65 illumination (Iwasaki Eye Colour arc lamp), a tripod in a 90° in relation to the butterfly's wing surface and at the same distance.

### IMAGE PROCESSING AND ANALYSES

The images were processed using a toolbox in the imaging software ImageJ (Rasband 1997), in which each photograph was linearized and normalized with regards to a gray standard (Stevens et al. 2007; Troscianko and Stevens 2015). Image data was mapped to the visual sensitivity of the relevant visual system using an image calibration and analysis toolbox, based on mathematically mapping from camera sensitivity to animal sensitivity (Stevens et al. 2007; Pike 2011; Troscianko and Stevens 2015). Predicted photon catch values were obtained for each colour, using the entire patch, applying spectral sensibility of each cone type of the blue tit (*Cyanistes caeruleus*) for the UV‐sensitive vision (Hart et al. 2000), peafowl (*Pavo cristatus*) for the Violet‐sensitive vision (Hart 2002), and *Heliconius erato* (Briscoe et al. 2010; McCulloch et al. 2016).

The color patches chosen were orange and yellow for *Hel/Hmm* (“rayed” pattern), red and yellow for *Hed/Hmr* (“postman” pattern), red and white for *Hen/Hmp* and white for *Hs/Hc*. We did not measure the iridescent blue dorsal and red ventral patterns in *Hs/Hc* comparisons. Although this pattern element differed between the two comimics, there were considerable differences in color and shape that made color capture difficult for our camera set‐up. Black areas of the wings were also not analyzed because values for these regions were consistently very low and uninformative for *Heliconius* races. To determine how well *Heliconius* comimic colors are matched, chromatic, and achromatic contrasts were quantified according to the receptor noise model developed by Vorobyev and Osorio (1998. We calculated achromatic contrast using bird double cones sensitivity. To account for receptor noise, a Weber fraction value of 0.05 was used for the most frequent cone type, as has been used in other models of bird and butterfly color vision (Vorobyev and Osorio 1998; Briscoe et al. 2010). Relative proportions of cone types were used to calculate chromatic contrast for the blue tit: LW = 1, MW = 0.99, SW = 0.71, UV = 0.37 (Hart et al. 2000), for peafowl: LW = 0.95, MW = 1, SW = 0.86, V = 0.45 (Hart 2002), and for *H. erato*: females, LW = 1, B = 0.17, UV2 = 0.076, UV1 = 0.086 and males, LW = 1, B = 0.2, UV2 = 0.13 (McCulloch et al. 2016). In *Heliconius*, it is not clear how the presence of red filtering pigments might influence color perception, therefore both possible wavelength sensitivities of the LW photoreceptors were used separately; Red‐LW (λmax = 600 nm) and Green‐LW (λmax = 555 nm) (Zaccardi et al. 2006; McCulloch et al. 2016).

The degree of discriminability between two colors is expressed in “just‐noticeable‐differences” (JND), based on a model of color distance that predicts that color contrasts result from a set of noise‐limited opponent color channels (Vorobyev and Osorio 1998). Normally, a JND of less than 3.00 should be difficult to discriminate in natural light conditions, whereas larger values allow increasingly easy discrimination (Siddiqi et al. 2004; Olsson et al. 2015). JND values were calculated for all pairwise comparisons within comimics and within conspecifics, separated by color and side of the wing, for *Heliconius*, UVS and VS vision models. For *Heliconius* vision, JND values within conspecifics were calculated only between *erato* clade wings, as the existing visual data is from *H. erato* eyes and the melpomene/cydno clade has a different retinal mosaic (McCulloch et al. 2017).

### UV MATING EXPERIMENT

To investigate whether the UV reflectance of natural butterflies affects mate preference, a mate choice test was carried out under natural sun light conditions inside a shaded cage. Adult males of *H. erato demophoon* were collected around Soberanía National Park, Panama, and kept in insectary facilities in Gamboa, Panama.

Butterfly wing models were made with wings dissected from *H. erato demophoon* and *H. melpomene rosina* female bodies and glued to adhesive black tape. The adhesive tape kept the wings together in an open wing position but also allowed movement of the model in a simulated flight, following methodology of earlier studies (Jiggins et al. 2004; Estrada and Jiggins 2008). To block UV, a sunscreen (Soltan© Invisible SPF30) cover was spread over the colored region of one pair, covering the red and yellow bands on both sides (UV‐). By covering the wing color bands using transparent sunscreen, UV reflectance was removed without changing the color, which we confirmed with UV photograph using the methodology described above. Sunscreen also covered the black part of the wings of the other pair, that is not covering any color, to control for smell (UV+).

Forty‐one males were used to test their response to models with UV blocked of the two different species inside a cage (2 × 1 × 2 m). Prior to experimental use, males were acclimated to the cage environment for at least 24 hours. Each male was tested twice and always offered the choice of two females: *H. erato* UV+ versus *H. melpomene* UV+, or *H. erato* UV– versus *H. melpomene* UV–. The models were placed 1 m apart, fixed on the ends of zip‐ties attached to a PVC pipe suspended between two metal bars. The PVC pipe was manipulated so that the models simulate butterfly flight (Jiggins et al. 2001; Finkbeiner et al. 2014).

Each pair of models was presented for 30 min to a single male, starting at the first sign of activity by the male. When a male flew toward the model to within a distance of 15 cm, the behavior was recorded as “approach,” and when a male came flying close to the model in a hovering or circling behavior, the behavior was recorded as “courtship” (Jiggins et al. 2001, 2004). Two replicate 30 min observation periods were carried out for each comparison and replicates were combined with total of 1 hour for analysis.

### STATISTICAL ANALYSES

First, we used the average of the pairwise JND values of each individual, compared to comimics and to conspecifics, which were grouped by comimic species, color, side of the wing and visual system. Then, to test whether differences between comimics were significantly higher than between conspecifics, we compared JNDs between comimics against JNDs between conspecifics using analysis of variance (one‐way ANOVA). To account for the fact that the same individuals were used for multiple analyses, that is comimics versus conspecifics in each color, side, and vision, individuals were set as random factor in the ANOVAs. Normality tests showed that JND data were not normally distributed; therefore the data was transformed to normality using square‐root transformation before statistical analyses. Raw (untransformed) JND data was plotted to illustrate the results. To evaluate mate choice experiments, a weighted binomial GLM was used to compare *H. erato* male proportion of successes of “approach” and “courtship attempts” toward its comimic *H. melpomene* female and to evaluate interaction between treatments, where the weight was the number of total successes and fails of each individual. All statistical calculations were processed with the packages *stats* and *ggplot2* in the software R 3.2.1 (R Core Team 2015).

## Results

### PREDATOR VISION SENSITIVITY

We amplified the SWS1 fragment sequence from all eight bird species (Table 1). Some of the sequences could be confirmed with previous studies that used same species, genus, or family (Ödeen and Håstad 2003, 2013). The Black‐tailed Trogon, Blue‐crowed Motmot, and Slaty Antshrike all had UVS visual system, while the remaining species had VS visual systems (Table 1).

**Table 1:** Predicted type of vision in examined bird species

				aa seq 84–94											
Order	Family	Species	Common Name			**86**				**90**			**93**		Type
Trogoniformes	Trogonidae	*Trogon melanurus*	Black‐tailed Trogon	F	I	**F**	C	V	F	**S**	V	F	**T**	V	UVS
Coraciiformes	Momotidae	*Momotus momota*	Blue‐crowed Motmot	F	I	**F**	C	S	F	**S**	V	F	**T**	V	UVS
Piciformes	Bucconidae	*Malacoptila panamensis*	White‐whiskered Puffbird	F	I	**S**	C	I	F	**S**	V	F	**T**	V	VS
Piciformes	Galbulidae	*Galbula ruficauda*	Rufous‐tailed Jacamar	L	M	**C**	C	I	F	**S**	V	F	**T**	V	VS
Passeriformes	Thamnophilidae	*Thamnophilus atrinucha*	Slaty Antshrike	F	M	**C**	C	I	F	**C**	I	F	**T**	V	UVS
Passeriformes	Tyrannidae	*Pitangus sulphuratus*	Great Kiskadee	F	M	**C**	C	I	F	**S**	V	F	**T**	V	VS
Passeriformes	Tyrannidae	*Mionectes oleagineus*	Ochre‐bellied Flycatcher	F	M	**C**	C	I	F	**S**	V	F	**T**	V	VS
Passeriformes	Tyrannidae	*Myiarchus panamensis*	Panama Flycatcher	F	M	**C**	C	I	F	**S**	V	F	**T**	V	VS

SWS1 amino acid sequences for the eight potential avian predators, showing sites from 84 to 94. In bold, sites 86, 90, and 93 are shown as sites where mutations are responsible for spectral tuning according to Wilkie et al. (2000). See Table S1 for GenBank accession numbers.

### COLOUR MIMICRY CONTRASTS TO AVIAN VISION

**Figure 1:**
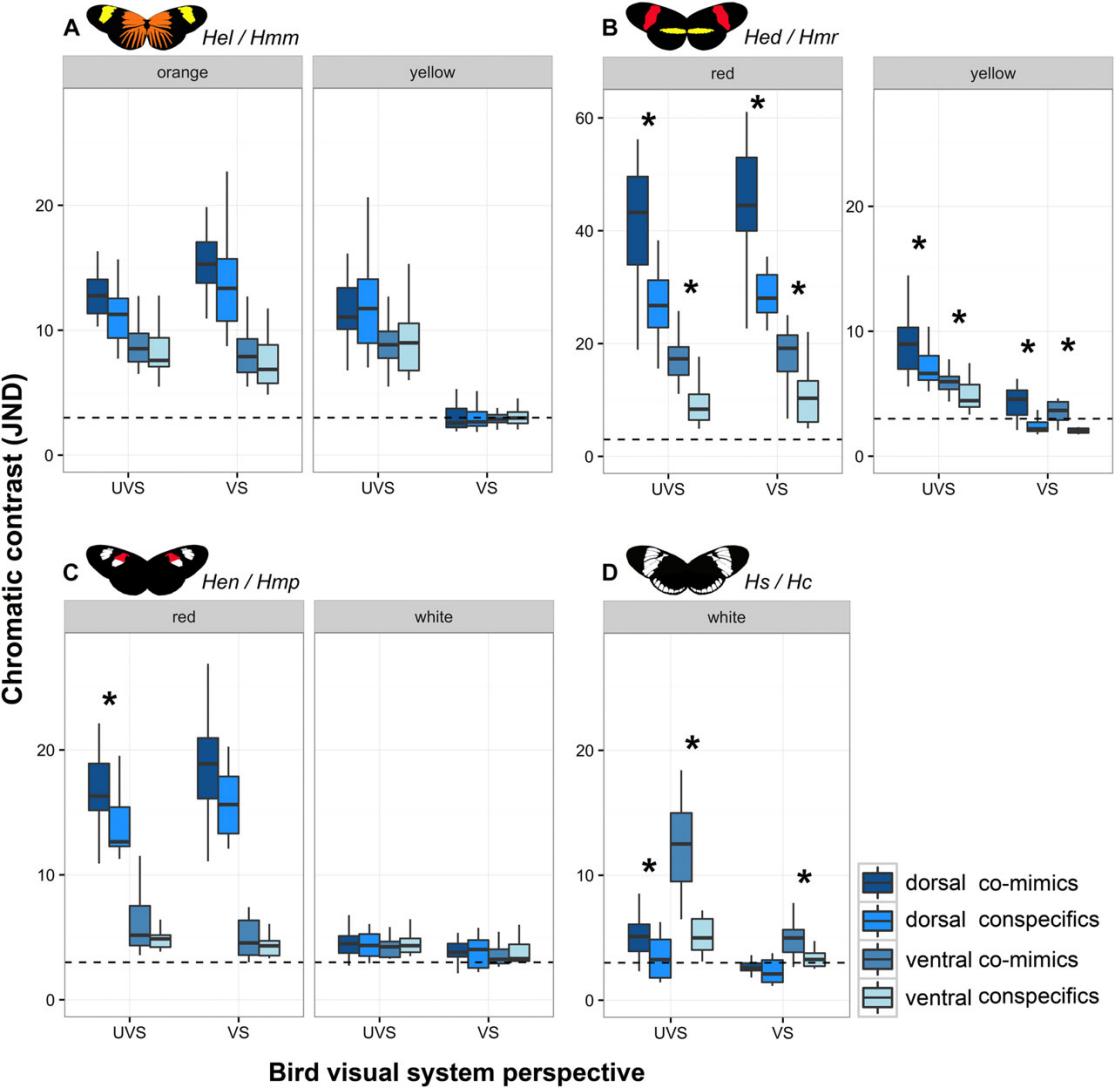
Chromatic comparison of color patches between conspecific and comimic specimens. Butterfly pictures illustrate comimics’ colors and patterns. Box plots show UVS and VS avian visual system JNDs between comimics and between conspecifics in each color and wing side: (A) *H. erato lativitta* and *H. melpomene malleti* (*Hel/Hmm*); (B) *H. e. demophoon* and *H. m. rosina* (*Hed/Hmr*); (C) *H. e. notabilis* and *H. m. plesseni* (*Hen/Hmp*); (D) *H. sapho* and *H. cydno* (*Hs/Hc*). Values > 3 JND denote an increasing ability to discriminate colours, whereas values ≤ 3 JND are generally difficult to distinguish (dashed line = 3). Box plots show median, upper, and lower quartile, maximum and minimum. Asterisks (*) show comimic JNDs that are statistically higher than conspecific JNDs (*P* < 0.05, see Table S2). Note that high JND values for red *Hed/Hmr* is due to the deep red hue giving rise to extreme relative values between wavelengths.

We used photography to compare each of the color patches between the pairs of comimic species. For both the *Hel/Hmm* and *Hen/Hmp* mimicry rings there were many JND values that were greater than the threshold of discrimination, especially for the UVS bird visual system (Fig. 1A and C). Butterflies in these mimicry pairs were more similar when observed by the VS visual system, where pairwise JNDs for white and yellow colors were close to the perception threshold. Nonetheless, in none of these comparisons was there any evidence for significantly greater JNDs in comparisons between comimics as compared to within conspecifics (Table S2). This indicates that, despite considerable individual level variation, there was no informative information between the comimics that could be used by predators to distinguish comimic pairs.

In contrast, for both the *Hed/Hmr* and *Hs/Hc* mimicry rings, there was evidence for significant differences between comimics that might be perceptible to predators (Fig. 1; Table S2). This was the case for red, yellow, and white patterns, especially with UVS visual systems (Fig. 1B and D). Under the VS visual system, there were significant differences (Fig. 1; Table S2) but in some cases these were not far above the discrimination threshold and may therefore not have much relevance in the wild (Fig. 1). JND values for red color comparisons were surprisingly high for two reasons. First, the deep red hue contrasts very markedly with the extremely low short‐ and middle‐wave reflectance, giving extreme relative values, and second there is also considerable variation in the red color between individuals according to their age (Dell'Aglio et al. 2017). Achromatic contrasts did not show significant differences between conspecifics and comimics to both vision systems, with the exception of the ventral red patch in *Hen/Hmp* (Table S3).

### COLOUR MIMICRY CONTRASTS THROUGH HELICONIUS VISION

**Figure 2:**
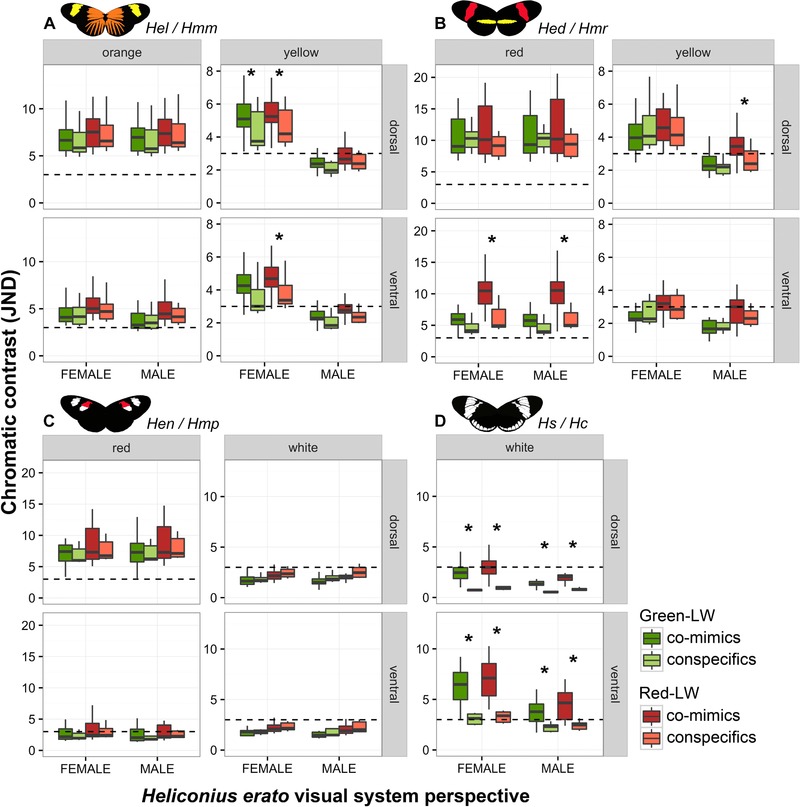
Chromatic comparison of colour patches between conspecific and comimic specimens. Butterfly pictures illustrate comimics’ colors and patterns. Box plots show *Heliconius erato* female and male visual system JNDs, using Green‐LW and Red‐LW sensitivities, between comimics and between conspecifics in each color and wing side: (A) *H. erato lativitta* and *H. melpomene malleti* (*Hel/Hmm*); (B) *H. e. demophoon* and *H. m. rosina* (*Hed/Hmr*); (C) *H. e. notabilis* and *H. m. plesseni* (*Hen/Hmp*); (D) *H. sapho* and *H. cydno* (*Hs/Hc*). Values > 3 JND denote an increasing ability to discriminate colours, whereas values ≤ 3 JND are generally difficult to distinguish (dashed line = 3). Box plots show median, upper, and lower quartile, maximum and minimum. Asterisks (*) show comimic JNDs that are statistically higher than conspecific JNDs (*P* < 0.05, see Table S4).

We used the recently published *H. erato* male and female visual models to examine how the same mimetic butterflies appear to conspecifics. Once again, similar to the bird vision models, both red and orange colours showed high JND values in the comparisons but these were mostly not significantly different between conspecifics and comimics. Only the red ventral pattern of the *Hed/Hmr* mimicry ring showed significant differences that might indicate a consistent difference between comimics (Table S4). It is worth noting that the Red‐LW sensitivity increases difference perception between comimics for red ventral and yellow dorsal for males in *Hed/Hmr* (Fig. 2B).

In contrast, yellow and white colors commonly showed greater differences between comimics than conspecifics, especially to the *H. erato* female visual system (Table S4). In particular the yellow band of the *Hel/Hmm* mimicry ring showed strong and significant differences in the female but not the male visual system (Fig. 2A, Table S4). White colors in the *Hs/Hc* mimicry ring, similar to the pattern seen for the bird visual system, were significantly different between comimics and conspecifics for all comparisons (Table S4). However, the JND values were only above the discrimination threshold for the ventral side of the wing (Fig. 2D).

### UV LIGHT AS A CUE IN SPECIES RECOGNITION

**Figure 3:**
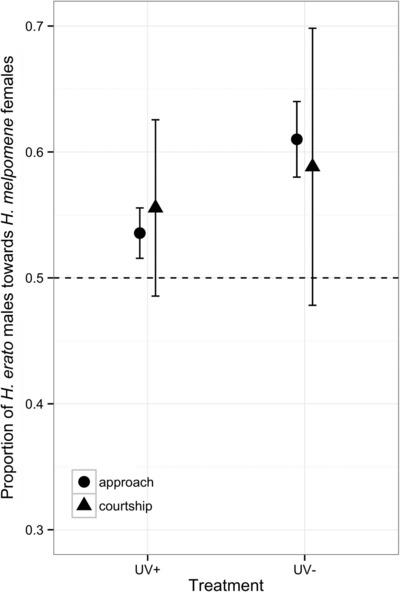
Males approach more frequently their comimic in the absence of UV. Proportion of *Heliconius erato* males to perform approach (circles) and courtship (triangles) behavior toward their comimic female *H. melpomene* over their own species female in two treatments, UV+ and UV– (mean ± SE).

Across our 41 males, we recorded 709 approaches and 62 courtships. The *H. erato* males approached *H. melpomene* females more frequently than their conspecifics in the UV‐ treatment showing that the absence of UV in wing coloration led to maladaptive male choice (*z* = 4.967, *P* < 0.001, Fig. 3). There was no difference in courtship behavior between species (*z* = 1.024, *P* = 0.306). Furthermore, there was a significant interaction between species and treatments for approach behaviour (*z* = –2.719, *P* = 0.006) but not for courtship attempts (*z* = –0.327, *P* = 0.743). Although males might be expected to approach females of their own species more than comimics, we found that approaches to the two species were close to random in the +UV treatment (Fig. 3).

## Discussion

Here, we have shown that despite using similar pigments and having nearly identical patterns, there are spectral differences that potentially could permit *Heliconius* and their avian predators to distinguish between comimics. Their colors contain information that can selectively signal to both potential avian predators and potential mates. However, we found no compelling evidence that these differences are private or unique to the butterfly visual system. Together our findings highlight the fact that visual system evolution between *Heliconius* and their predators is even more complex than originally imagined. These differences in perception may play a role in conspecific recognition for courtship and mating, and we have provided support for this hypothesis using behavioral assays. Furthermore, we have also characterized the visual system of potential *Heliconius* predators found in Panama.

We have shown that among eight species of birds found in the Canal Zone area of Panama, both UVS and VS visual systems are represented. Our findings confirm the expected SWS1 sequences of one species that have been studied previously, *Momotus momota* (Ödeen and Håstad 2013). In other cases related taxa have been studied before and our results are consistent with expectations, that is the two genera *Trogon* and *Myiarchus*, and the families Bucconidae and Tyrannidae (Ödeen and Håstad 2003). The one exception is *Thamnophilus atrinucha* in which mutations confer UVS vision, which differs from other VS vision Thamnophilidae species studied previously (Seddon et al. 2010). From 21 avian orders studied earlier, the SWS1 gene for avian color vision shifted between VS and UVS at least 14 times, such that color vision is not highly conserved between families (Ödeen and Håstad 2013). All of these species are potential butterfly predators, although the only species that have been tested experimentally with *Heliconius* are Jacamars and Flycatchers which both have the VS vision (Chai 1986; Pinheiro 1996).

In the light of this information on predator visual systems, there is some limited support for the hypothesis of a cryptic channel of communication available to the butterflies (Bybee et al. 2012). We have found that the dorsal yellow colors on the *Hel/Hmm* pair are not distinguishable to either bird visual system, but visual modeling suggests that female *H. erato* would be able to distinguish these patterns. The fact that the female visual system specifically is able to distinguish yellow patches is also consistent with a role in sexual behavior. This therefore supports the earlier suggestion that yellow colors might act as species‐specific cues in *Heliconius* (Bybee et al. 2012; Finkbeiner et al. 2017). However, this pattern is far from general, as among our samples the yellow band of the *Hed/Hmr* mimicry pair is perhaps more readily distinguishable by birds, in particular those with a UVS visual system, than it is for butterflies.

Similarly, in the case of red and white colors there is little evidence for a private channel of communication. In both avian visual systems, *Hed/Hmr* red and yellow bands and *Hs/Hc* white comimic chromatic contrasts were significantly higher than those between conspecifics. However, there was also considerable variation between conspecifics, and we recently showed considerable variation in the red color between individuals according to their age (Dell'Aglio et al. 2017). Therefore, there is some potential for predators to perceive differences between these species. However, given the precision of mimicry in *Heliconius* in other aspects such as wing pattern and flight, it seems likely that the colours are sufficiently similar that predators generalize between the comimics.

Previous research on the color patterns of two polymorphic Müllerian mimic butterflies, *Heliconius numata* and *Melinaea*, has revealed that small differences in contrast between comimics can be perceived more effectively by butterflies than birds, with these contrasts being computed between yellow/orange against the black of the wing (Llaurens et al. 2014). Another comimic pair analyzed was *Heliconius sara* and *Mimoides pausanias*, and although they have wing differences, they look similar under avian violet vision (Thurman and Seymoure 2016). Similarly, population level variation indicates that *Heliconius timareta* has evolved to match the local coloration of its comimic *H. melpomene amaryllis* (Mérot et al. 2016). A further study on Batesian mimics found differences between the sexes and wing surfaces, with females being better mimics and the dorsal side having better resemblance to mimic models (Su et al. 2015). Other study systems have also investigated warning signal evolution and utilized vision modelling such as in *Anolis* lizards (Fleishman et al. 2016), ladybirds (Arenas and Stevens 2017), and tiger moths (Henze et al. 2018).

Our work benefited from recent advances in understanding *Heliconius* vision, and in particular the discovery of sexual dimorphism in the visual system of *H. erato* (McCulloch et al. 2016, 2017). This dimorphism is likely to play a role in conspecific recognition. For example, in the *Hel/Hmm* mimicry pair, the yellow dorsal and ventral, JND values for comparisons between comimics were significantly higher than for conspecifics in the female vision model, but not in that for males. This perhaps suggests that the presence of an extra UV opsin in females might allow them to better distinguish conspecific mates. *Heliconius* are also unusual in that their visual sensitivity is shifted to red in the LW photoreceptor by the presence of filtering pigments. Our modeling suggests that this shift makes some colors more distinguishable as compared to the Green‐LW sensitivity, for example *Hed/Hmr* red ventral. It has been suggested that differences in certain parts of the eye may arise for specific visual tasks, and these LW photoreceptors that contain red filtering pigments may be adapted for mate choice (Briscoe and Chittka 2001). Sexual dimorphism in *H. erato* eyes may therefore help discrimination between comimics, possibly avoiding confusion between close mimetic colour patterns, and could therefore represent an example of coadaptation between signals and sensory systems.

Our modeling suggests that in some cases butterfly visual systems can better distinguish ventral as compared to dorsal colors. In *Heliconius*, it seems likely that dorsal colors might have evolved through selection for aposematism as antipredator protection, while ventral surfaces are selected for sexual signaling. During courtship behavior males show off their ventral side while hovering over the female, which may make it easier for females to recognize conspecific males (Klein and de Araújo 2010). In other butterflies there is clear evidence for signal partitioning between dorsal and ventral wings (Rutowski et al. 2010). In *Bicyclus*, dorsal wing characters are involved in sexual signaling while the eyespots in the ventral wing have a role in predator avoidance (Robertson and Monteiro 2005; Oliver et al. 2009; De Bona et al. 2015). Also, blue *Morpho* butterflies show intense iridescent blue coloration on the dorsal side that is involved in males flight patrolling, whereas on the ventral side cryptic colors and big eyespots may have been selected against visual predators (DeVries et al. 2010).

The role of UV signals in sexual selection is also supported by our behavioral experiment. Removing UV reflectance influences mate choice. It is notable that our behavioral experiments considered the responses of males, which are known from previous work to respond strongly to color cues. Although recent data shows that females express the extra UV sensitive opsin, both sexes express the UV1 opsin gene, so are expected to be able to detect UV cues. It is nonetheless rather surprising that *H. erato* males seem to prefer wings of *H. melpomene*, perhaps due to an absence of other pheromonal and behavioral cues in our experiments. As seen in a previous mating study with *H. erato*, our results have also shown that UV might be less important for courtship than it is for approach behavior (Finkbeiner et al. 2017). However, our work contributes to previous studies showing that UV light influences mating behavior in butterflies, notably in the Pieridae, which can visually discriminate between sexes using UV cues (Silberglied and Taylor Jr. 1973; Kemp 2008), as well as *Bicyclus*, in which small UV‐reflective spots played a role in female choice (Robertson and Monteiro 2005).

Our key conclusions are based on models of vision and, like all models, these can have some limitations in their ability to reproduce the complex vision of animals. Color measures are convenient because they offer an intuitive means of analyzing phenotypes that may not be accurately represented with human vision, and give us valuable insights into biological questions. The use of digital cameras to model reflectance spectra is widely and increasingly used for making these measures (Stevens et al. 2007; Pike 2011; Troscianko and Stevens 2015). Photography is often better in controlling for light conditions, accounting for larger color areas and spatial variation, and for angles of measurements than spectrometry, because it captures the whole scene (Lovell et al. 2005; Spottiswoode and Stevens 2010; Stevens et al. 2014). In fact, it has been shown to produce more accurate data than spectrometry in recent work (del Valle et al. 2018) and modeling of animal receptor responses is highly accurate with appropriate camera methods compared to spectrometry (Stevens and Cuthill 2006; Pike 2011; Troscianko and Stevens 2015). However, we emphasize that these models need to be fully tested in the future using behavioral data to determine whether an observer can effectively discriminate pairs of colors in a manner as predicted by the models. Our results show insights into color discrimination and its role in communication in *Heliconius*, but we hope that they can be further verified with behavioral tests.

In summary, it is clear that avian predators and conspecifics may often perceive coloration in *Heliconius* butterflies differently. In general, UVS birds can detect differences between comimics and conspecifics better than VS birds, perhaps suggesting that *Heliconius* mimicry is more effective against VS predators. This is consistent with the fact that the two most widely studied predators of *Heliconius*, Jacamars and Tyrannidae both have a VS visual system. Furthermore, there is evidence that sexually dimorphic vision in *H. erato* might confer an advantage to females in perceiving differences between comimics. Moreover, *Heliconius* males use UV signals for mate choice, indicating that conflicting forces of natural and sexual selection affect visual signals, both reducing cost of confusion in courtship and maintaining the advantages of warning coloration. Apart from aposematic colouration, *Heliconius* butterflies have other adaptations that might also help to reduce risk of predation, such as levels of toxicity, antipredator behavior, and chemical cues that might also act to enhance the protective benefits of mimicry.

Associate Editor: E. Derryberry

Handling Editor: P. Tiffin

## Supporting information


**Table S1**. Biorepository ID for bird samples used archived in the Smithsonian Tropical Research Institute Cryological Collection in Panama and accession number for the SWS1 opsin gene at GenBank.
**Table S2**. One‐way ANOVA (with individuals as random factors) results for chromatic JND comparisons between co‐mimics and conspecifics for Figure 1.
**Table S3**. One‐way ANOVA (with individuals as random factors) results for achromatic JND comparisons between co‐mimics and conspecifics.
**Table S4**. One‐way ANOVA (with individuals as random factors) results for chromatic JND comparisons between co‐mimics and conspecifics for Figure 2.
**Figure S1**. Higher UV reflectance is perceived on the ventral side of the yellow and red bands.
