# Supplementary material for: The appearance of mimetic Heliconius butterflies to predators and conspecifics
Source: Evolution. 2018 Sep 5;72(10):2156–66. doi: 10.1111/evo.13583 (PMC6221148; doi:10.1111/evo.13583)

**The appearance of mimetic *Heliconius* butterflies to predators and conspecifics**

**Supporting Information**

**Table S1.** Biorepository ID for bird samples used archived in the Smithsonian Tropical Research Institute Cryological Collection in Panama and accession number for the SWS1 opsin gene at GenBank.

|  |  |  |  |
| --- | --- | --- | --- |
| Species | Common name | Biorepository ID | GenBank nº |
| *Trogon melanurus* | Black-tailed Trogon | BBT19878 | MH745970 |
| *Momotus momota* | Blue-crowed Motmot | BBT18191 | MH745971 |
| *Malacoptila panamensis* | White-whiskered Puffbird | BBT10765 | MH745972 |
| *Galbula ruficauda* | Rufous-tailed Jacamar | KDKC0583 | MH745973 |
| *Thamnophilus atrinucha* | Slaty Antshrike | BBT11166 | MH745974 |
| *Pitangus sulphuratus* | Great Kiskadee | BBT17670 | MH745975 |
| *Mionectes oleagineus* | Ochre-bellied Flycatcher | BBT10964 | MH745976 |
| *Myiarchus panamensis* | Panama Flycatcher | BBT17572 | MH745977 |
|  |  |  |  |

**Table S2.** One-way ANOVA (with individuals as random factors) results for chromatic JND comparisons between co-mimics and conspecifics for Figure 1. *N,* number of individuals; ANOVA results, *F*, *P* and *df* (degrees of freedom); Error (residual values), *MS* (mean square) and *df* (degrees of freedom). Results are shown for UVS and VS avian vision system in each co-mimic pair, colour patch and side of the wing. *Hel/Hmm*: *H. erato lativitta* and *H. melpomene malleti*. *Hed/Hmr*: *H. e. demophoon* and *H. m. rosina*. *Hen/Hmp*: *H. e. notabilis* and *H. m. plesseni*. *Hs/Hc*: *H. sapho* and *H. cydno*. Number in bold indicate P < 0.05.

|  |  |  |  |  |  |  |  |  |  |  |  |  |  |  |  |
| --- | --- | --- | --- | --- | --- | --- | --- | --- | --- | --- | --- | --- | --- | --- | --- |
| Co-mimics | |  |  |  | Avian vision system | | | | | | | | | | |
|  | Colour |  |  |  | UVS | | | | |  | VS | | | | |
|  |  | Wing side | |  |  |  |  | error | |  |  |  |  | error | |
|  |  |  | N |  | F | P | df | MS | df |  | F | P | df | MS | df |
| Hel / Hmm | |  |  |  |  |  |  |  |  |  |  |  |  |  |  |
|  | Yellow | dorsal | 56 |  | 0.13 | 0.717 | 1 | 0.196 | 53 |  | 0.03 | 0.870 | 1 | 0.057 | 53 |
|  |  | ventral | 56 |  | 0.09 | 0.771 | 1 | 0.154 | 53 |  | 0.06 | 0.802 | 1 | 0.035 | 53 |
|  | Orange | dorsal | 56 |  | 3.64 | 0.061 | 1 | 0.203 | 53 |  | 1.99 | 0.164 | 1 | 0.372 | 53 |
|  |  | ventral | 56 |  | 2.19 | 0.145 | 1 | 0.113 | 53 |  | 2.82 | 0.099 | 1 | 0.154 | 53 |
| Hed / Hmr | |  |  |  |  |  |  |  |  |  |  |  |  |  |  |
|  | Yellow | dorsal | 40 |  | 6.30 | **0.017** | 1 | 0.138 | 37 |  | 31.72 | **1.98E-06** | 1 | 0.088 | 37 |
|  |  | ventral | 40 |  | 8.24 | **0.007** | 1 | 0.068 | 37 |  | 29.06 | **4.20E-06** | 1 | 0.058 | 37 |
|  | Red | dorsal | 40 |  | 25.04 | **1.4E-05** | 1 | 0.696 | 37 |  | 22.00 | **3.66E-05** | 1 | 0.659 | 37 |
|  |  | ventral | 40 |  | 52.30 | **1.4E-08** | 1 | 0.269 | 37 |  | 20.25 | **0.0001** | 1 | 0.670 | 37 |
| Hen / Hmp | |  |  |  |  |  |  |  |  |  |  |  |  |  |  |
|  | White | dorsal | 40 |  | 0.05 | 0.828 | 1 | 0.062 | 37 |  | 0.23 | 0.634 | 1 | 0.074 | 37 |
|  |  | ventral | 40 |  | 0.70 | 0.409 | 1 | 0.058 | 37 |  | 0.53 | 0.469 | 1 | 0.077 | 37 |
|  | Red | dorsal | 40 |  | 4.34 | **0.044** | 1 | 0.301 | 37 |  | 1.58 | 0.217 | 1 | 0.679 | 37 |
|  |  | ventral | 40 |  | 3.04 | 0.090 | 1 | 0.143 | 37 |  | 1.82 | 0.185 | 1 | 0.147 | 37 |
| Hs / Hc |  |  |  |  |  |  |  |  |  |  |  |  |  |  |  |
|  | White | dorsal | 40 |  | 10.01 | **0.003** | 1 | 0.1844 | 37 |  | 3.92 | 0.055 | 1 | 0.059 | 37 |
|  |  | ventral | 40 |  | 74.97 | **2.0E-10** | 1 | 0.195 | 37 |  | 17.58 | **0.0002** | 1 | 0.071 | 37 |
|  |  |  |  |  |  |  |  |  |  |  |  |  |  |  |  |

**Table S3.** One-way ANOVA (with individuals as random factors) results for achromatic JND comparisons between co-mimics and conspecifics. *N,* number of individuals; ANOVA results, *F*, *P* and *df* (degrees of freedom); Error (residual values), *MS* (mean square) and *df* (degrees of freedom). Results are shown for UVS and VS avian vision system in each co-mimic pair, colour patch and side of the wing. *Hel/Hmm*: *H. erato lativitta* and *H. melpomene malleti*. *Hed/Hmr*: *H. e. demophoon* and *H. m. rosina*. *Hen/Hmp*: *H. e. notabilis* and *H. m. plesseni*. *Hs/Hc*: *H. sapho* and *H. cydno*. Number in bold indicate P < 0.05.

|  |  | |  |  |  |  |  |  |  |  |  |  |  |  |  |  |
| --- | --- | --- | --- | --- | --- | --- | --- | --- | --- | --- | --- | --- | --- | --- | --- | --- |
| Co-mimics | | |  |  |  | Avian vision system | | | | | | | | | | |
|  | Colour | |  |  |  | UVS | | | | |  | VS | | | | |
|  |  | | Wing side | |  |  |  |  | error | |  |  |  |  | error | |
|  |  | |  | N |  | F | P | df | MS | df |  | F | P | df | MS | df |
| Hel / Hmm | | |  |  |  |  |  |  |  |  |  |  |  |  |  |  |
|  | | Yellow | dorsal | 56 |  | 1.35 | 0.251 | 1 | 0.161 | 53 |  | 1.24 | 0.270 | 1 | 0.166 | 53 |
|  | |  | ventral | 56 |  | 1.06 | 0.308 | 1 | 0.258 | 53 |  | 1.07 | 0.305 | 1 | 0.2631 | 53 |
|  | | Orange | dorsal | 56 |  | 2.13 | 0.151 | 1 | 0.107 | 53 |  | 1.61 | 0.210 | 1 | 0.104 | 53 |
|  | |  | ventral | 56 |  | 0.47 | 0.497 | 1 | 0.063 | 53 |  | 0.46 | 0.501 | 1 | 0.068 | 53 |
| Hed / Hmr | |  |  |  |  |  |  |  |  |  |  |  |  |  |  |  |
|  | | Yellow | dorsal | 40 |  | 0.01 | 0.939 | 1 | 0.108 | 37 |  | 0.01 | 0.930 | 1 | 0.104 | 37 |
|  | |  | ventral | 40 |  | 5.01 | **0.031** | 1 | 0.245 | 37 |  | 4.46 | **0.042** | 1 | 0.234 | 37 |
|  | | Red | dorsal | 40 |  | 1.09 | 0.303 | 1 | 0.289 | 37 |  | 0.08 | 0.784 | 1 | 0.196 | 37 |
|  | |  | ventral | 40 |  | 0.08 | 0.785 | 1 | 0.159 | 37 |  | 0.80 | 0.378 | 1 | 0.174 | 37 |
| Hen / Hmp | | |  |  |  |  |  |  |  |  |  |  |  |  |  |  |
|  | White | | dorsal | 40 |  | 0.93 | 0.341 | 1 | 0.083 | 37 |  | 0.13 | 0.721 | 1 | 0.066 | 37 |
|  |  | | ventral | 40 |  | 0.10 | 0.757 | 1 | 0.226 | 37 |  | 0.67 | 0.420 | 1 | 0.203 | 37 |
|  | Red | | dorsal | 40 |  | 2.38 | 0.132 | 1 | 0.151 | 37 |  | 6.23 | **0.017** | 1 | 0.149 | 37 |
|  |  | | ventral | 40 |  | 9.02 | **0.005** | 1 | 0.183 | 37 |  | 7.88 | **0.008** | 1 | 0.168 | 37 |
| Hs / Hc |  | |  |  |  |  |  |  |  |  |  |  |  |  |  |  |
|  | White | | dorsal | 40 |  | 0.17 | 0.684 | 1 | 0.114 | 37 |  | 0.18 | 0.671 | 1 | 0.118 | 37 |
|  |  | | ventral | 40 |  | 0.78 | 0.384 | 1 | 0.231 | 37 |  | 0.92 | 0.343 | 1 | 0.232 | 37 |
|  |  | |  |  |  |  |  |  |  |  |  |  |  |  |  |  |

**Table S4.** One-way ANOVA (with individuals as random factors) results for chromatic JND comparisons between co-mimics and conspecifics for Figure 2. *N,* number of individuals; ANOVA results, *F*, *P* and *df* (degrees of freedom); Error (residual values), *MS* (mean square) and *df* (degrees of freedom). Results are shown for *Heliconius* *erato* female and male vision system in each co-mimic pair, colour patch, side of the wing and LW sensitivity. *Hel/Hmm*: *H. erato lativitta* and *H. melpomene malleti*. *Hed/Hmr*: *H. e. demophoon* and *H. m. rosina*. *Hen/Hmp*: *H. e. notabilis* and *H. m. plesseni*. *Hs/Hc*: *H. sapho* and *H. cydno*. Number in bold indicate P ≤ 0.05.

|  |  |  |  |  |  |  |  |  |  |  |  |  |  |  |  |  |
| --- | --- | --- | --- | --- | --- | --- | --- | --- | --- | --- | --- | --- | --- | --- | --- | --- |
| Co-mimics | |  |  |  |  | Butterfly vision system | | | | | | | | | | |
|  | Colour |  |  |  |  | Females | | | | |  | Males | | | | |
|  |  | Wing side | |  |  |  |  |  | error | |  |  |  |  | error | |
|  |  |  | LW | N |  | F | P | df | MS | df |  | F | P | df | MS | df |
| Hel / Hmm | |  |  |  |  |  |  |  |  |  |  |  |  |  |  |  |
|  | Yellow | dorsal | Green | 42 |  | 5.62 | **0.023** | 1 | 0.071 | 39 |  | 3.15 | 0.084 | 1 | 0.034 | 39 |
|  |  |  | Red | 42 |  | 4.83 | **0.034** | 1 | 0.061 | 39 |  | 1.26 | 0.269 | 1 | 0.037 | 39 |
|  |  | ventral | Green | 42 |  | 4.04 | 0.052 | 1 | 0.084 | 39 |  | 2.52 | 0.121 | 1 | 0.044 | 39 |
|  |  |  | Red | 42 |  | 4.53 | **0.040** | 1 | 0.039 | 39 |  | 3.01 | 0.091 | 1 | 0.038 | 39 |
|  | Orange | dorsal | Green | 42 |  | 0.69 | 0.411 | 1 | 0.138 | 39 |  | 0.82 | 0.370 | 1 | 0.161 | 39 |
|  |  |  | Red | 42 |  | 0.57 | 0.456 | 1 | 0.165 | 39 |  | 0.68 | 0.414 | 1 | 0.189 | 39 |
|  |  | ventral | Green | 42 |  | 0.001 | 0.974 | 1 | 0.061 | 39 |  | 0.02 | 0.899 | 1 | 0.089 | 39 |
|  |  |  | Red | 42 |  | 1.03 | 0.317 | 1 | 0.075 | 39 |  | 0.49 | 0.489 | 1 | 0.104 | 39 |
| Hed / Hmr | |  |  |  |  |  |  |  |  |  |  |  |  |  |  |  |
|  | Yellow | dorsal | Green | 30 |  | 1.03 | 0.320 | 1 | 0.082 | 27 |  | 0.64 | 0.430 | 1 | 0.048 | 27 |
|  |  |  | Red | 30 |  | 0.25 | 0.620 | 1 | 0.082 | 27 |  | 6.00 | **0.021** | 1 | 0.072 | 27 |
|  |  | ventral | Green | 30 |  | 0.96 | 0.337 | 1 | 0.035 | 27 |  | 0.22 | 0.642 | 1 | 0.036 | 27 |
|  |  |  | Red | 30 |  | 0.57 | 0.458 | 1 | 0.048 | 27 |  | 1.04 | 0.317 | 1 | 0.064 | 27 |
|  | Red | dorsal | Green | 30 |  | 0.01 | 0.921 | 1 | 0.185 | 27 |  | 0.01 | 0.914 | 1 | 0.219 | 27 |
|  |  |  | Red | 30 |  | 1.72 | 0.200 | 1 | 0.305 | 27 |  | 1.73 | 0.200 | 1 | 0.366 | 27 |
|  |  | ventral | Green | 30 |  | 3.45 | 0.074 | 1 | 0.102 | 27 |  | 3.90 | 0.059 | 1 | 0.104 | 27 |
|  |  |  | Red | 30 |  | 22.34 | **6.36E-05** | 1 | 0.168 | 27 |  | 17.30 | **0.0003** | 1 | 0.203 | 27 |
| Hen / Hmp | |  |  |  |  |  |  |  |  |  |  |  |  |  |  |  |
|  | White | dorsal | Green | 30 |  | 0.47 | 0.500 | 1 | 0.027 | 27 |  | 3.70 | 0.065 | 1 | 0.035 | 27 |
|  |  |  | Red | 30 |  | 0.90 | 0.352 | 1 | 0.031 | 27 |  | 3.06 | 0.092 | 1 | 0.036 | 27 |
|  |  | ventral | Green | 30 |  | 0.39 | 0.538 | 1 | 0.029 | 27 |  | 1.04 | 0.318 | 1 | 0.046 | 27 |
|  |  |  | Red | 30 |  | 0.39 | 0.539 | 1 | 0.0384 | 27 |  | 1.06 | 0.313 | 1 | 0.053 | 27 |
|  | Red | dorsal | Green | 30 |  | 0.002 | 0.967 | 1 | 0.418 | 27 |  | 0.05 | 0.819 | 1 | 0.474 | 27 |
|  |  |  | Red | 30 |  | 0.03 | 0.860 | 1 | 0.494 | 27 |  | 0.002 | 0.968 | 1 | 0.539 | 27 |
|  |  | ventral | Green | 30 |  | 1.04 | 0.318 | 1 | 0.086 | 27 |  | 0.69 | 0.413 | 1 | 0.094 | 27 |
|  |  |  | Red | 30 |  | 0.54 | 0.470 | 1 | 0.142 | 27 |  | 0.33 | 0.568 | 1 | 0.152 | 27 |
| Hs / Hc | |  |  |  |  |  |  |  |  |  |  |  |  |  |  |  |
|  | White | dorsal | Green | 30 |  | 41.54 | **6.6E-07** | 1 | 0.072 | 27 |  | 29.70 | **9.13E-06** | 1 | 0.039 | 27 |
|  |  |  | Red | 30 |  | 35.43 | **2.4E-06** | 1 | 0.100 | 27 |  | 22.90 | **5.42E-05** | 1 | 0.071 | 27 |
|  |  | ventral | Green | 30 |  | 28.07 | **1.37E-05** | 1 | 0.120 | 27 |  | 11.27 | **0.002** | 1 | 0.093 | 27 |
|  |  |  | Red | 30 |  | 35.59 | **2.32E-06** | 1 | 0.123 | 27 |  | 19.86 | **0.0001** | 1 | 0.097 | 27 |
|  |  |  |  |  |  |  |  |  |  |  |  |  |  |  |  |  |

**Figure S1.** Higher UV reflectance is perceived on the ventral side of the yellow and red bands. Photographed species; left, RGB photo; right, UV photo: *Heliconius erato lativitta*, (a) dorsal, (c) ventral; *H. melpomene malleti*, (b) dorsal, (d) ventral; *H. e. demophoon*, (f) dorsal, (h) ventral; *H. m. rosina*, (g) dorsal, (i) ventral; *H. e. notabilis*, (j) dorsal, (l) ventral; *H. m. plesseni*, (k) dorsal, (m) ventral; *H. sapho*, (n) dorsal, (p) ventral; *H. cydno*, (o) dorsal, (q) ventral. These are uncalibrated photographs and were taken at the same distance.


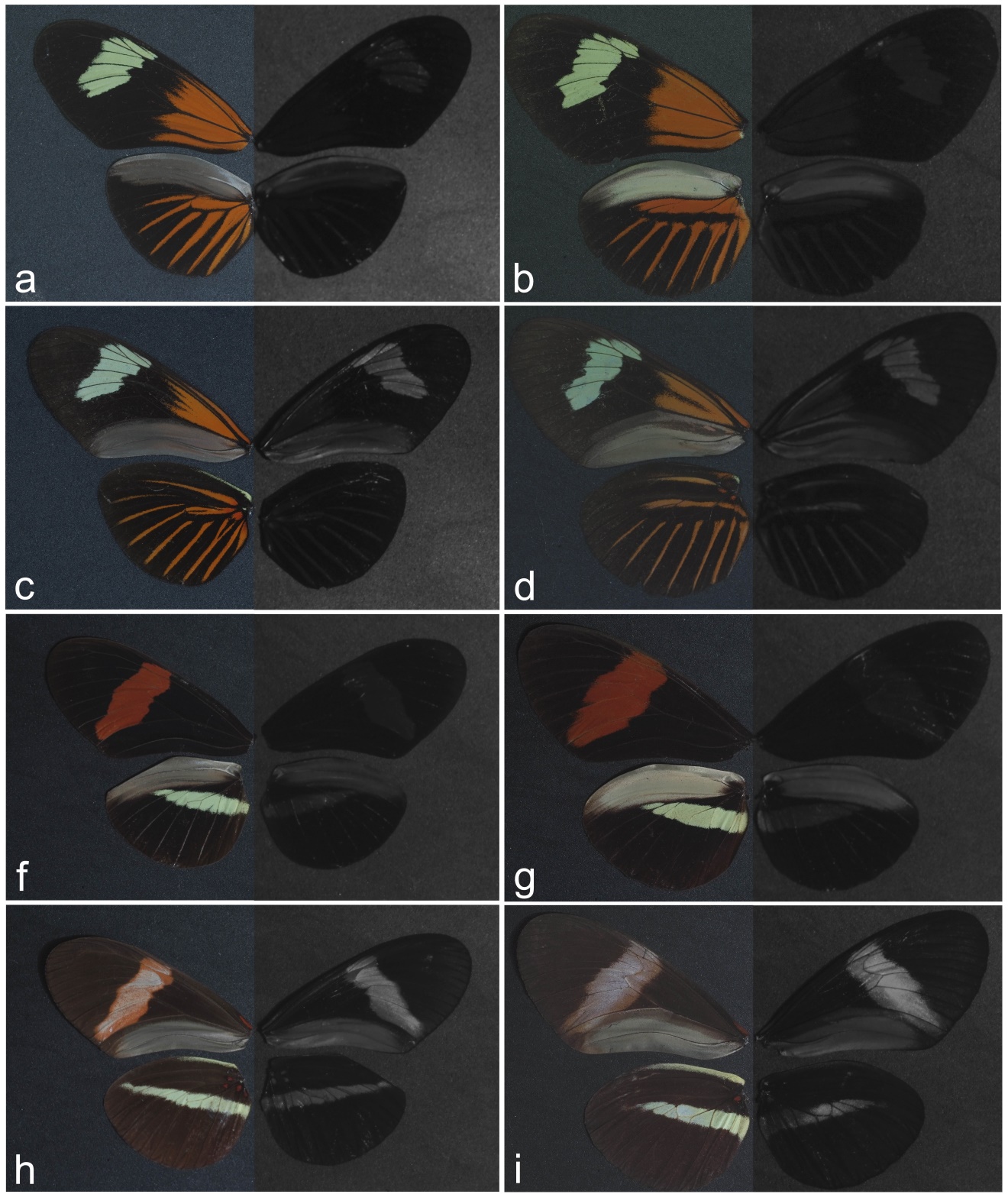


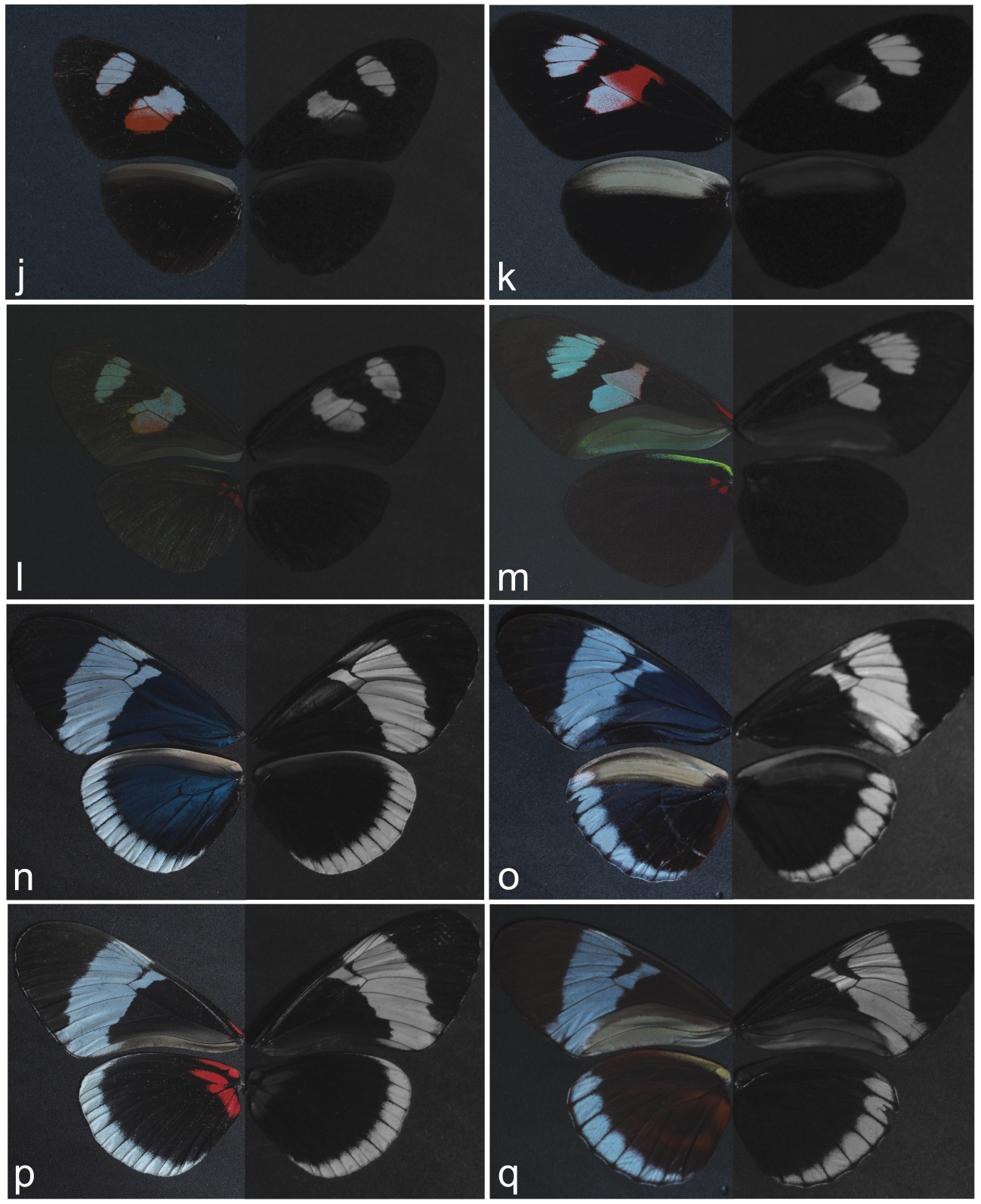

Supplement: Supplementary file 1 — Table S1. Biorepository ID for bird samples used archived in the Smithsonian Tropical Research Institute Cryological Collection in Panama and accession number for the SWS1 opsin gene at GenBank. Table S2. One‐way ANOVA (with individuals as random factors) results for chromatic JND comparisons between co‐mimics and conspecifics for Figure 1. Table S3. One‐way ANOVA (with individuals as random factors) results for achromatic JND comparisons between co‐mimics and conspecifics. Table S4. One‐way ANOVA (with individuals as random factors) results for chromatic JND comparisons between co‐mimics and conspecifics for Figure 2. Figure S1. Higher UV reflectance is perceived on the ventral side of the yellow and red bands. [file EVO-72-2156-s001.docx]
